# Enhancement of Low-field Magnetoresistance in Self-Assembled Epitaxial La_0.67_Ca_0.33_MnO_3_:NiO and La_0.67_Ca_0.33_MnO_3_:Co_3_O_4_ Composite Films via Polymer-Assisted Deposition

**DOI:** 10.1038/srep26390

**Published:** 2016-07-06

**Authors:** Meng Zhou, Yuling Li, Il Jeon, Qinghua Yi, Xuebin Zhu, Xianwu Tang, Haiyan Wang, Ling Fei, Yuping Sun, Shuguang Deng, Yutaka Matsuo, Hongmei Luo, Guifu Zou

**Affiliations:** 1 College of Physics, Optoelectronics and Energy & Collaborative Innovation Center of Suzhou Nano Science and Technology, Soochow University, Suzhou 215006, China; 2 Department of Chemical and Materials Engineering, New Mexico State University, New Mexico 88003, USA; 3 Department of Chemistry, School of Science, The University of Tokyo, 7-3-1 Hongo, Bunkyo-ku, Tokyo 113-0033, Japan; 4 Key Laboratory of Materials Physics, Institute of Solid State Physics; High Magnetic Field Laboratory, Chinese Academy of Science, Hefei 230031, China; 5 Department of Electrical and Computer Engineering, Texas A&M University, Texas 77843, USA

## Abstract

Polymer-assisted deposition method has been used to fabricate self-assembled epitaxial La_0.67_Ca_0.33_MnO_3_:NiO and La_0.67_Ca_0.33_MnO_3_:Co_3_O_4_ films on LaAlO_3_ substrates. Compared to pulsed-laser deposition method, polymer-assisted deposition provides a simpler and lower-cost approach to self-assembled composite films with enhanced low-field magnetoresistance effect. After the addition of NiO or Co_3_O_4_, triangular NiO and tetrahedral Co_3_O_4_ nanoparticles remain on the surface of La_0.67_Ca_0.33_MnO_3_ films. This results in a dramatic increase in resistivity of the films from 0.0061 Ω•cm to 0.59 Ω•cm and 1.07 Ω•cm, and a decrease in metal-insulator transition temperature from 270 K to 180 K and 172 K by the addition of 10%-NiO and 10%-Co_3_O_4_, respectively. Accordingly, the maximum absolute magnetoresistance value is improved from −44.6% to −59.1% and −52.7% by the addition of 10%-NiO and 10%-Co_3_O_4_, respectively. The enhanced low-field magnetoresistance property is ascribed to the introduced insulating phase at the grain boundaries. The magnetism is found to be more suppressed for the La_0.67_Ca_0.33_MnO_3_:Co_3_O_4_ composite films than the La_0.67_Ca_0.33_MnO_3_:NiO films, which can be attributed to the antiferromagnetic properties of the Co_3_O_4_ phase. The solution-processed composite films show enhanced low-field magnetoresistance effect which are crucial in practical applications. We expect our polymer-assisted deposited films paving the pathway in the field of hole-doped perovskites with their intrinsic colossal magnetoresistance.

The discovery of intrinsic colossal magnetoresistance (MR) in hole-doped perovskites with a formula, La_1−x_A_x_MnO_3_ (A = Ca, Sr, and Ba) has established their potential applications in memory devices and magnetic sensors^1^,^2^,^3^,^4^,^5^,^6^,^7^. However, practical application of the colossal MR is limited by the high magnetic field requirement. Therefore, it is very important to achieve high MR at a low magnetic field. Many reported works have suggested that the regions of structural disorder generated during the film deposition, grain boundaries in short, are the dominant factors in achieving the enhanced low-field magnetoresistance (LFMR). Also introducing a second phase, especially the insulating phase, into La_1−x_A_x_MnO_3_ films has been considered an effective approach for engineering the grain boundaries^8^,^9^,^10^,^11^,^12^,^13^,^14^,^15^,^16^,^17^,^18^,^19^,^20^,^21^,^22^. For example, LCMO:ZrO_2_ (LCMO: La_0.67_Ca_0.33_MnO_3_) composite films produced by the sol-gel method achieved a MR value of −31% at 77 K under 0.1 T^21^. Also, −25% of MR at 93 K under 1.15 T was observed in LCMO:15% V_2_O_5_ composite fabricated by a two-step solid-state reaction^22^ and a pronounced LFMR of −23.9% at 10 K in a field of 0.5 T was achieved in LSMO:ZnO (LSMO: La_0.67_Sr_0.33_MnO_3_) composite film^14^. Finally, a tunable and enhanced LFMR of −30% at 154 K under 0.1 T was observed in a LSMO:ZnO nanocomposite film grown by pulsed-laser deposition (PLD). Most of these reported composite films were, however, prepared by either complicated multiple-steps or costly high-vacuum processes.

Solution deposition method has advantages of low cost, easy setup, and coating of large area as it does not require vacuum-based expensive capital equipment^23^. Here we report self-assembled LCMO:NiO and LCMO:Co_3_O_4_ composite films epitaxially grown by polymer-assisted deposition (PAD) technique which demonstrate enhanced LFMR. Facile and cost-effective PAD method utilizes metal salts dissolved in an aqueous polymer solution^24^,^25^,^26^,^27^,^28^,^29^. Amount of metal cations bound to the polymer and the solution viscosity are directly related to amount of the polymer used. Thus, desired stoichiometry ratio could easily be controlled by mixing different metal-polymer precursor solutions with corresponding metal molar ratios. Thickness of the film could also be controlled by adjusting concentration of the solution or spin-coating rate. The properties of NiO or Co_3_O_4_ added LCMO composite films were investigated in both electronic and magnetic perspectives to assess LFMR effects. Under a magnetic field of 3 T, MR values of −44.6% at 255 K for LCMO, −59.1% at 180 K for LCMO:10%-NiO, and −52.7% at 172 K for LCMO:10%-Co_3_O_4_ were achieved, respectively. This revealed that the composite films possess high LFMR and their effect was enhanced even further with the addition of 10%-NiO or 10%-Co_3_O_4_ insulating phases of the grain boundaries.

## Results

### Lattice Parameters of Self-epixatially Grown LCMO

**Figure 1:**
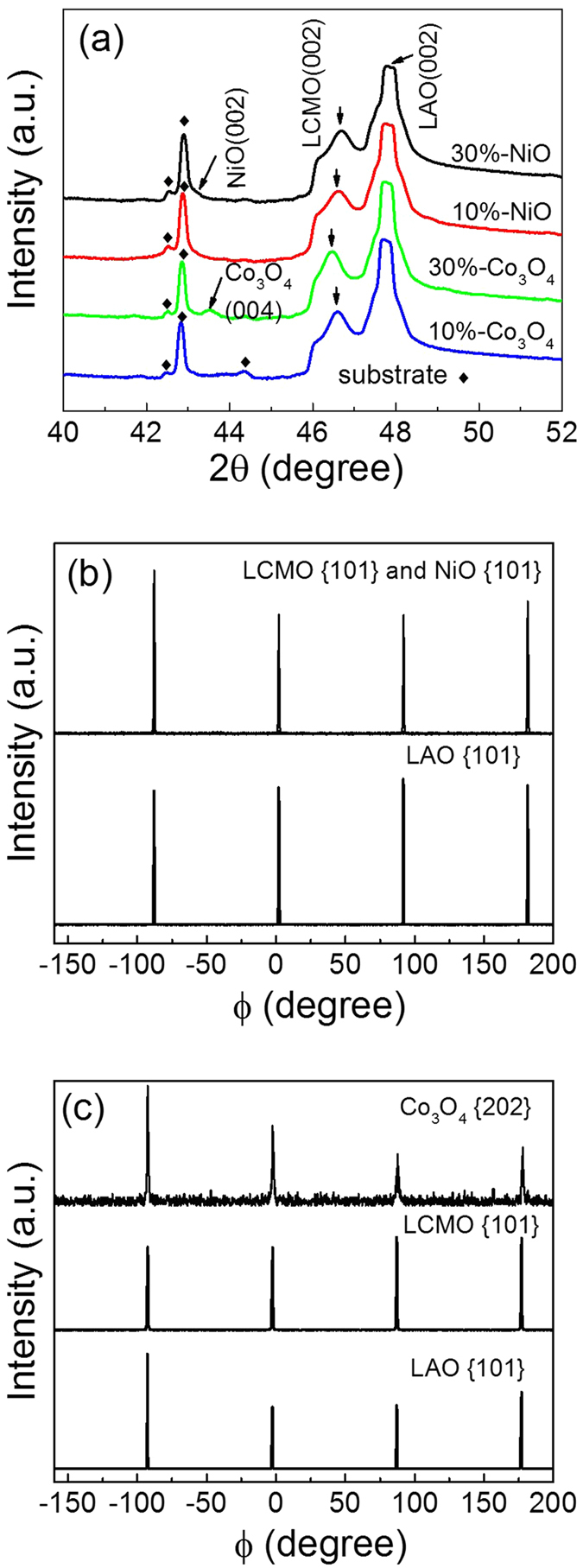
XRD patterns of (**a**) θ-2θ scan of LCMO:30%-NiO, LCMO:10%-NiO, LCMO:30%-Co_3_O_4_ and LCMO:10%-Co_3_O_4_ films grown on LAO substrate with additional peaks ¨ from substrates; (**b**) ϕ-scans of LCMO:30%-NiO from (101) reflections of LCMO, NiO, and LAO; and (**c**) ϕ-scans LCMO:30%-Co_3_O_4_ from (101) reflections of LCMO and LAO, and (202) reflection of Co_3_O_4_.

Lattice parameters and epitaxial growth were analyzed by in-plane and out-of-plane X-ray diffraction (XRD). XRD data of LCMO:Co_3_O_4_ and LCMO:NiO films grown on LaAlO_3_ (LAO) substrate are shown in Fig. 1. θ–2θ scans in Fig. 1a confirm the introduction of NiO (002) and Co_3_O_4_ (004) along with the (002) peaks for LCMO and LAO. The appearance of (00l) only series peaks of the films indicates that the films are highly textured along the c-axis, which is perpendicular to the substrate surface. It is noted that additional peaks appear from the single crystal substrates, apparently the substrates contain impurities. The similar peaks from LAO substrates were found in other reference as well^30^. The out-of-plane lattice parameters of LCMO:10%- and 30%-Co_3_O_4_ were calculated from the LCMO (002) peak to be 3.898 Å and 3.909 Å, respectively, while the out-of-plane lattice parameters of LCMO:10%- and 30%-NiO film were 3.897 Å and 3.891 Å, respectively. As expected, all the values were larger than that of the bulk LCMO (a = 3.858 Å for a pseudocubic perovskite unit cell). This elongation along the c-axis can be attributed to relatively larger lattice parameters of NiO (cubic structure with a = 4.177 Å) and Co_3_O_4_ (inverse spinel with a = 8.084 Å). According to the Poisson relationship, the in-plane lattice parameters are compressed when the out-of-plane parameters are expanded. Therefore, we know that the magnitude of the in-plane lattice parameter is in the following order, a (30%-Co_3_O_4_) <a (10%-Co_3_O_4_) ≈ a (10%-NiO) <a (30%-NiO). Figure 1b and c display the φ scans on reflections of LAO {101}, LCMO {101}, Co_3_O_4_ {202}, and NiO {101} of these composite films. The epitaxial growth of LCMO:Co_3_O_4_ or LCMO:NiO on LAO could be deduced from these aligned peaks. The {101} peaks of NiO seem to overlap the {101} peaks of LCMO, because the lattice parameter of NiO is close to that of LCMO. The heteroepitaxial relationships between the films (LCMO:Co_3_O_4_ and LCMO:NiO composite) and the substrate can be described as (001)_LCMO_//(002)_Co3O4_//(001)_LAO_, [101]_LCMO_//[202]_Co3O4_//[101]_LAO_, and (001)_LCMO_//(001)_NiO_//(001)_LAO_, [101]_LCMO_//[101]_NiO_//[101]_LAO_. Such epitaxial relationships are in accordance with the basal plane lattice parameters of LCMO (a = 3.858 Å), NiO (a = 4.177 Å), Co_3_O_4_ (a = 8.084 Å), and LAO (a = 3.79 Å). The lattice mismatch was calculated to be 1.8% between LCMO and LAO; 6.6% between Co_3_O_4_ and LAO, while 4.7% between Co_3_O_4_ and LCMO; 10.2% between NiO and LAO, and 8.2% between NiO and LCMO. Relatively low lattice mismatch enabled the epitaxial growths of LCMO and Co_3_O_4_, LCMO and NiO, LCMO:Co_3_O_4_ and LCMO:NiO composites on LAO.

### Morphology Investigations of the LCMO Composites

**Figure 2:**
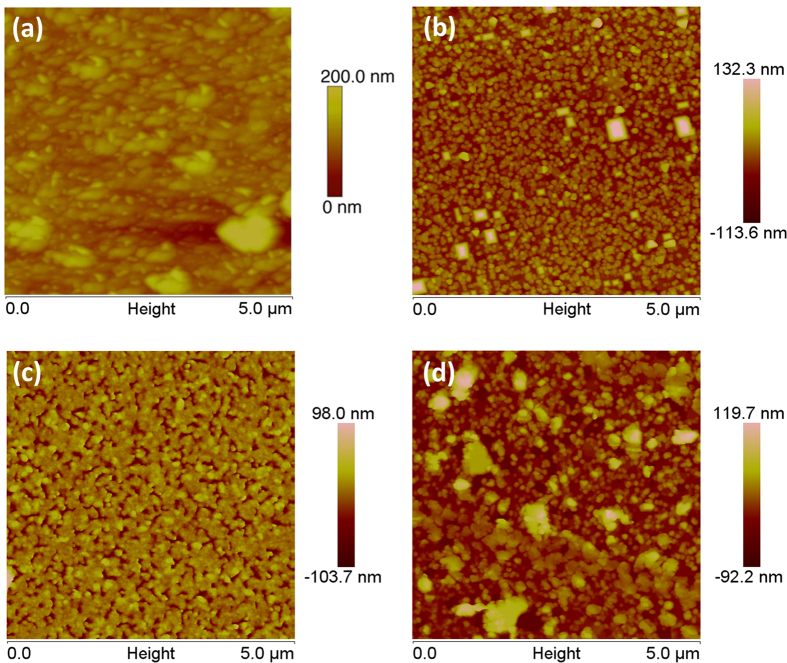
AFM images of (**a**) LCMO:30%-NiO; (**b**) LCMO:30%-Co_3_O_4_; (**c**) LCMO:10%-NiO; and (**d**) LCMO:10%-Co_3_O_4_ films grown on LAO substrate.

Morphology of the films was analyzed using atomic force microscopy (AFM), as shown in Fig. 2. Both the LCMO:30%-NiO and LCMO:30%-Co_3_O_4_ composite films showed the uniform surface with the root-mean-square (rms) surface roughness of around 15 nm for LCMO:30%-NiO and 17 nm for LCMO:30%-Co_3_O_4_. Triangular NiO and tetrahedral Co_3_O_4_ particles were visible on the continuous LCMO matrix surface. The surface of LCMO:10%-NiO is much smoother with rms of 3 nm, indicating that NiO particles are smaller, likely most particles are embedded in the LCMO matrix since they are not obvious on the surface. However, LCMO:10%-Co_3_O_4_ has larger Co_3_O_4_ particles with different shapes sitting on the surface and the composite has rms of 16 nm.

**Figure 3:**
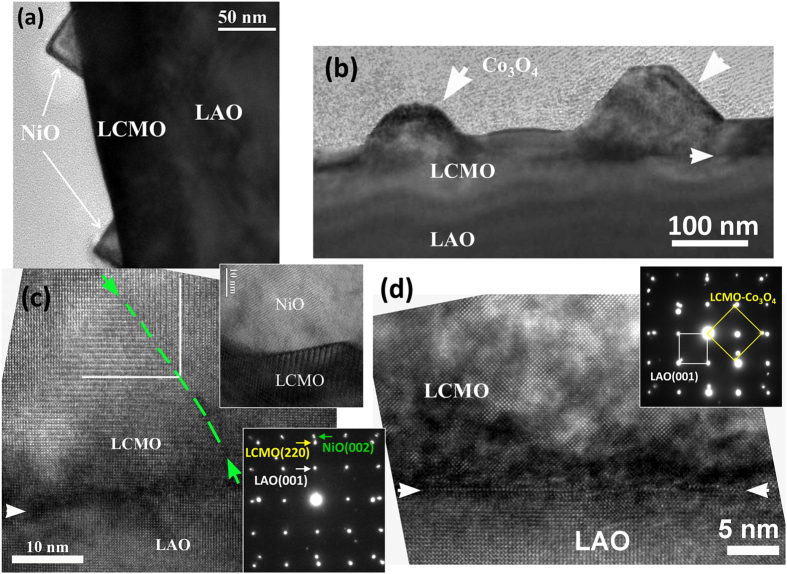
Bright-field TEM images of (**a**) LCMO:30%-NiO and (**b**) LCMO:30%-Co_3_O_4_ films grown on LAO substrates; cross-sectional high resolution TEM images of (**c**) LCMO:30%-NiO with inset image for NiO epitaxially grown with respect to LCMO matrix; and (**d**) LCMO:30%-Co_3_O_4_ on LAO substrates. The insets are the corresponding SAED patterns for each film (Yellow represents LCMO and LCMO-Co_3_O_4_, White represents LAO, and green represent NiO).

The microstructures of these composite films were also studied by cross-sectional TEM and high resolution TEM (HRTEM). From Fig. 3a,b, in addition to the substrates, two phases are clearly observed in the films as triangular NiO nanoparticles (50–100 nm) and tetrahedral Co_3_O_4_ nanoparticles (100–200 nm) on the surface of LCMO. They are consistent with the morphologies shown in the AFM images. The cross-sectional HRTEM images are displayed in Fig. 3c,d. We can see that the interface between the LCMO phase and the substrates are cleanly divided without intermixing. The interface between NiO and LCMO, as shown in the inset, clearly comfirms the epitaxial grown NiO with LCMO matrix. The corresponding selected area electron diffraction (SAED) patterns further confirm the epitaxial growth of LCMO:NiO and LCMO:Co_3_O_4_ on LAO by showing the distinct diffraction dots of LCMO, NiO (or Co_3_O_4_), and LAO from the SAED patterns; the epitaxial relationships between the composite films and the LAO substrate are identical to the XRD analysis.

### Electrical Properties of the LCMO Composites

**Figure 4:**
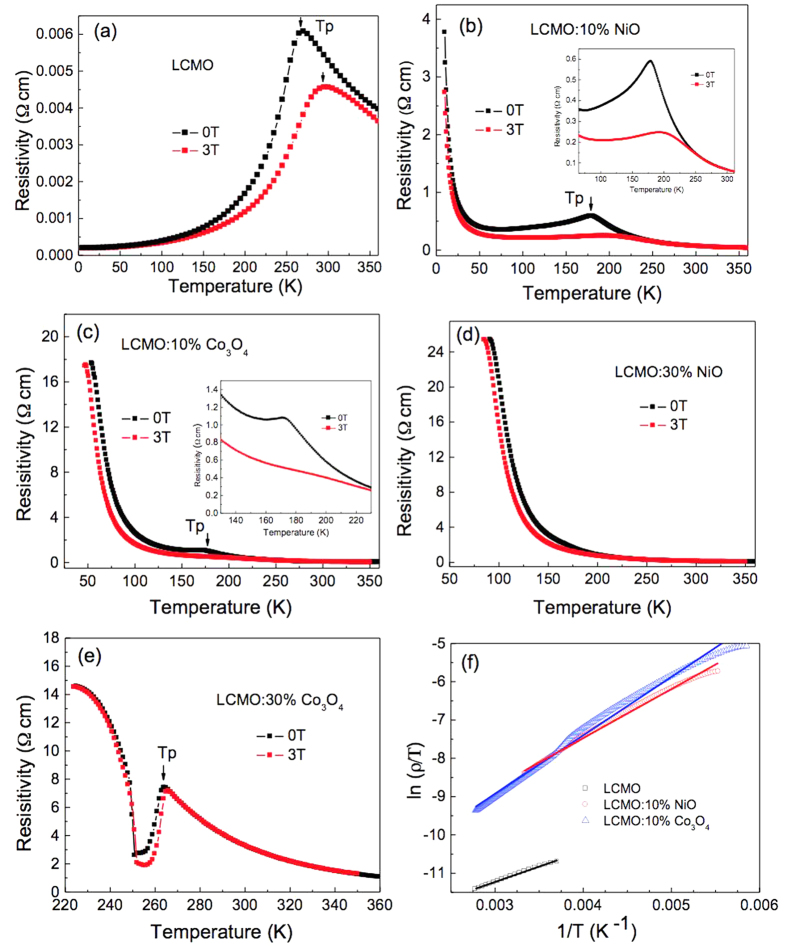
Temperature dependent resistivity (ρ) of (**a**) LCMO, (**b**) LCMO:10%-NiO, (**c**) LCMO:10% Co_3_O_4_, (**d**) LCMO:30%-NiO, and (**e**) LCMO:30%-Co_3_O_4_ films at applied magnetic fields of 0 T and 3 T. (**f**) Plots of adiabatic expression ln(ρ/T) vs 1/T for LCMO, LCMO:10%-NiO, and LCMO:10%-Co_3_O_4_ films at temperatures higher than T_P_ and zero magnetic field, with the best fits. The insets are magnifications of the curves around T_P_.

By studying temperature dependent resistivity (ρ) over temperature at different applied magnetic fields, different metal-insulator transition temperatures (T_P_s) were recorded from the composite films. In Fig. 4(a), a well-defined metal-insulator transition feature is displayed from the LCMO film, with the temperature of maximum resistivity at 270 K for 0 T and 295 K for 3 T. Nevertheless, unlike the single phase LCMO films, the composite films do not show metallic behaviors at lower temperatures. For the LCMO:10%-NiO and LCMO:10%-Co_3_O_4_ composite films in Fig. 4b,c, respectively, the transition peaks are much broader and less obvious. The T_P_s under zero field are 180 K for LCMO:10%-NiO and 172 K for LCMO:10%-Co_3_O_4_. Moreover, the peak resistivity of these two composite films (0.59 Ω•cm for LCMO:10%-NiO and 1.07 Ω•cm for LCMO:10%-Co_3_O_4_ at zero magnetic field) are much higher than that of the single phase LCMO film (0.0061 Ω•cm at zero magnetic field). These two observations can be attributed to the presence of a large number of the grain boundaries, increased disorder, as well as insulating phase-induced barriers to the electrical transport^14^,^31^,^32^,^33^. The introduced insulating phase at the grain boundary has been known to obstruct the magnetic spin alignment near the grain boundary region. Therefore, it increased the tunneling barrier height between the neighboring magnetic grains^13^,^34^,^35^.

As for the LCMO:30%-NiO and LCMO:30%-Co_3_O_4_ composite films shown in Fig. 4d,e, respectively, within the measured temperature range, LCMO:30%-NiO shows a semiconductor behavior without metal-insulator transition, whereas for LCMO:30%-Co_3_O_4_, the film manifests an interesting behavior. There is a clear metal-insulator transition at the T_P_ of 265 K in comparison to the other composite films; the resistivity increases sharply with the further temperature decrease. Also, the resistivity increases for the LCMO:30%-Co_3_O_4_ composite film in comparison to that of the LCMO:10%-Co_3_O_4_ composite film. This is because the metal-insulator transition was suppressed by the expansion of in-plane lattice parameter in LCMO^14^,^22^.

As mentioned above, the LCMO:30%-NiO composite film has the largest in-plane lattice constant and this led to the disappearance of metal-insulator transition. On the other hand, for the LCMO:30%-Co_3_O_4_ composite film, it has the smallest in-plane lattice constant. Therefore, even though the secondary phase of Co_3_O_4_ suppressed the metal-insulator transition, the compressed in-plane lattice constant enhanced the transition, leading to an enhanced T_P_ as shown in Fig. 4(e). The sharp increase in resistivity at low temperatures can be attributed to the possible localization originating from the large amount of Co_3_O_4_ secondary phase^36^,^37^. Several previous studies have shown that the behavior of paramagnetic phase in LCMO can be well described by the small-polaron transport^37^,^38^. For the charge transport by polarons, the resistivity is given by the equation 1:


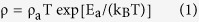


where ρ_a_ is resistivity coefficient, k_B_ is Boltzmann’s constant, and E_a_ is activation energy that is related to the polaron binding energy^13^. Therefore, we can have the expression 2:


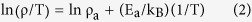


Figure 4f shows the plots of ln (ρ/T) vs 1/T and their linear fittings for the LCMO, LCMO:10%-NiO, and LCMO:10%-Co_3_O_4_ films at temperatures higher than T_P_ with zero magnetic field. The slope of the linear fitting line is proportional to the activation energy E_a_. This indicates the energy barrier height for the spin-dependent electron hopping at the grain boundaries^17^. Thus, we can patently see that the energy barrier height of the LCMO film increases with the addition of the second phase i.e., NiO and Co_3_O_4_. In addition, the ln (ρ/T) vs 1/T curves for the LCMO:10%-NiO, and LCMO:10%-Co_3_O_4_ films could not show good linear relation. It might be from the variable range hopping mechanism, low-temperature conduction in strongly disordered systems with localized charge-carrier states^39^.

### Magnetic Properties of the LCMO Composites and enhanced LFMR

**Figure 5:**
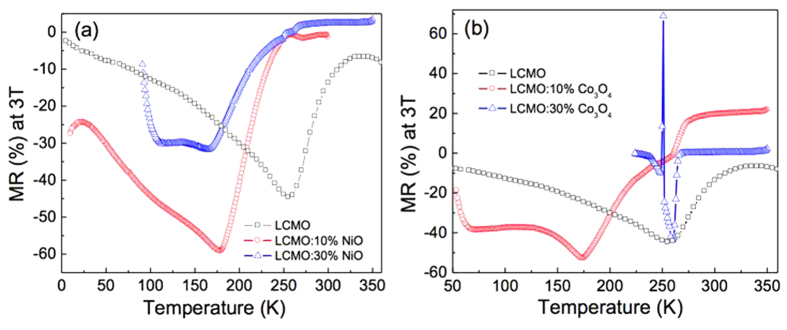
The calculated temperature dependent MR of (**a**) LCMO, LCMO:10%-NiO, and LCMO:30%-NiO films; (**b**) LCMO, LCMO:10%-Co_3_O_4_, and LCMO:30%-Co_3_O_4_ films at the applied magnetic field of 3 T.

Enhancement in LFMR is a unique feature and important advantage of the PAD-produced composite films. Figure 5 compares the temperature dependent MR of single phase LCMO and the LCMO composite films with different percentages of NiO and Co_3_O_4_, which were calculated from the resistivity at magnetic fields of 0 T and 3 T using the equation, MR (%) = (ρ_H_ − ρ_0_)/ρ_0_ × 100%. For the composite films, MR curves have several small maxima at different temperatures, while the single phase LCMO film shows a more simplistic curve. This phenomenon may be due to phase inhomogeneity of the composite films^29^. The maximum MR values increased for the films with the 10% addition of either NiO or Co_3_O_4_: −44.6% at 255 K for LCMO, −59.1% at 180 K for LCMO:10%-NiO, and −52.7% at 172 K for LCMO:10%-Co_3_O_4_. These MR values are similar to those of other LCMO-based composite films prepared by PLD technique, but the temperature dependent resistivity behavior is different^37^,^40^,^41^,^42^. For the LCMO:30%-NiO film, the MR value was slightly lower than that of the single phase LCMO film, due to its larger expansion of the in-plane lattice parameter. Whereas, the LCMO:30%-Co_3_O_4_ film delivered a comparable MR value to that of the single phase LCMO film. It is worth noting that the T_P_ of LCMO:30%-Co_3_O_4_ film was the highest among the four composite samples.

**Figure 6:**
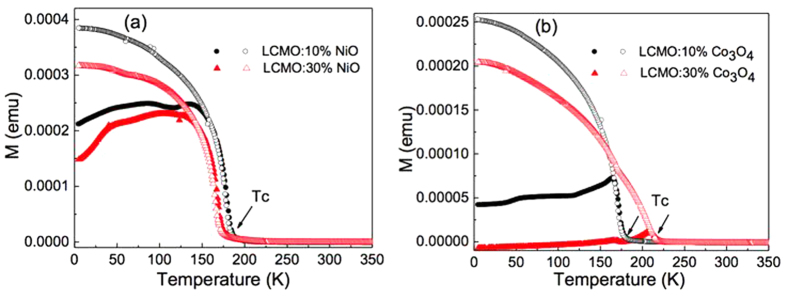
ZFC (closed symbols) and FC (open symbols) of magnetization-temperature (M-T) curves measured at 100 Oe for (**a**) LCMO:NiO and (**b**) LCMO:Co_3_O_4_ composite films.

The nature of the magnetically ordered state was investigated by classical zero-field cooled (ZFC) and field cooled (FC) cycles. Figure 6 presents the temperature-dependent magnetization curves measured under an applied field of 100 Oe for the four composite films. All the curves show a similar trend and the FC curves coincides with the ZFC curves at the high temperatures, while the FC curves differ from the ZFC curves in the lower temperature region^37^,^43^. It should be mentioned that Curie temperature (T_C_) of the LCMO:Co_3_O_4_ films increases with the addition of Co_3_O_4_, whereas for the LCMO:NiO films, T_C_ decreases with the addition of NiO. The enhanced T_C_ for the LCMO:Co_3_O_4_ films can be attributed to the decreased in-plane lattice parameter with the addition of Co_3_O_4_. On the contrary, the decreased T_C_ for the LCMO:NiO films should be attributed to the increased in-plane lattice parameter with the addition of NiO. Moreover, the similar T_C_s for the LCMO:10%-NiO and LCMO:10%-Co_3_O_4_ owing to the similar in-plane lattice parameter further suggests that the magnetic properties as well as transport properties are dominantly controlled by the in-plane lattice parameter. In addition, the magnetism at low temperatures is clearly suppressed for the LCMO:Co_3_O_4_ composite films compared to that of the LCMO:NiO films, which can be explained by the antiferromagnetic properties of Co_3_O_4_ phase.

For a facile comparison, out-of-plane lattice parameter, T_P_ , ρ, MR, and T_C_ are summarized in Table 1. Difference between the LCMO:30%-Co_3_O_4_ and LCMO:30%-NiO films, and similarity between the LCMO:10%-NiO and LCMO:10%-Co_3_O_4_ films in out-of-plane lattice parameter can be seen. In addition, T_P_ and T_C_ show an opposite tendency to the in-plane lattice parameter. LCMO:10%-NiO and LCMO:10%-Co_3_O_4_ films show similar T_P_ and T_C_ because of the similar lattice parameters. Meanwhile, the resistivities are different due to distinct conducting properties of Co_3_O_4_ and NiO.

## Conclusion

In summary, we successfully demonstrated PAD method to fabricate LCMO:NiO and LCMO:Co_3_O_4_ epitaxial self-assembled composite films on LAO substrates. PAD method, which provides an alternative yet simpler approach to the growth of self-assembled composite films, is more cost effective compared to the conventional PLD methods. Moreover, the films produced by the PAD method showed enhancement in the LFMR effect which is crucial in the practical application, as demonstrated by the temperature dependent MR analysis. The maximum absolute MR values were further improved by the addition of NiO or Co_3_O_4_ due to introduced insulating phase at the grain boundaries. This is entails dramatic increase in resistivity and decrease in T_P_ with the addition of NiO and Co_3_O_4_. The temperature-dependent magnetization curve also revealed that T_C_ showed the same trend as T_P_ . More suppressed magnetism was observed from the LCMO:Co_3_O_4_ composite films than the LCMO:NiO films on account of the antiferromagnetic properties of the Co_3_O_4_ phase. The solution-processed epitaxial films with the enhanced LFMR effect presented here will bring a breakthrough in the field of hole-doped perovskites and their growth.

## Methods

### Sample preparation

To prepare the precursor solutions for the nanocomposite films, the individual metal-polymer aqueous solutions were prepared first by dissolving 2 g of corresponding metal salts (La(NO_3_)_3_∙6H_2_O, Ca(OH)_2_, MnCl_2_∙H_2_O, NiCl_2_, or CoCl_2_∙6H_2_O) into the polymer solution, which contained 4 g of polyethylenimine (PEI, 50 wt% in water, branched polymer, average Mn ~60,000 by GPC, average Mw ~750,000 by LS, Aldrich) and 2 g of ethylenediaminetetraacetic acid (EDTA, anhydrous, 99%, Aldrich) in 40 g of de-ionized (DI) water. These metal-polymer solutions were then filtered in an Amicon unit, which is designed to pass materials with molecular weight of less than 30,000 g/mol, to remove the unbound ions and concentrate the solutions. The concentrations of La^3+^, Ca^2+^, Mn^4+^, Co^3+^, and Ni^2+^ of the final solutions were determined as 125, 191, 148, 245, and 334 mM, respectively, by inductively coupled plasma atomic emission spectroscopy (ICP-AES). These as-prepared solutions were mixed in desired stoichiometric ratios of the single phase LCMO and composite LCMO:Co_3_O_4_ or LCMO:NiO (molar ratios of LCMO:Co_3_O_4_ or LCMO:NiO are 0.9:0.1 and 0.7:0.3) thin films. The mixed solutions were spin-coated onto LaAlO_3_ (001) substrates (http://www.mtixtl.com/) at 2,500 rpm for 30 s. The samples were heated at 550 °C for 2 h in flowing oxygen to remove polymer. Films of about 20 nm thickness were obtained from one spin-coat. It is noted that thicker films can be achieved by multiple spin-coats. For the final step, the films were annealed at 950 °C for 2 h in flowing oxygen to improve the crystallization and get epitaxial thin films.

### Sample Characterization

The crystal structure of the films was characterized by X-ray diffraction (XRD). The surface morphology and microstructures were analyzed by atomic force microscopy (AFM) and high resolution transmission electron microscopy (HRTEM). The temperature dependence of resistivity was measured by a Quantum Design Physical Property Measurement System (PPMS) along the film surface using a standard four-probe method, with the magnetic field applied normal to the film surface. Temperature-dependent magnetization was measured by a superconducting quantum interference device (SQUID) magnetometer.

## Additional Information

**How to cite this article**: Zhou, M. *et al*. Enhancement of Low-field Magnetoresistance in Self-Assembled Epitaxial La_0.67_Ca_0.33_MnO_3_:NiO and La_0.67_Ca_0.33_MnO_3_:Co_3_O_4_ Composite Films via Polymer-Assisted Deposition. *Sci. Rep.* **6**, 26390; doi: 10.1038/srep26390 (2016).

## Figures and Tables

**Table 1:** Summary of parameters of LCMO:NiO and LCMO:Co_3_O_4_ composite films prepared from PAD method.

**Sample**	**Out-of-plane lattice parameter [Å]**	**T**_**P**_**[K]**	**ρ** **[Ω•cm]**	**MR [%]**	**T**_**C**_**[K]**
LCMO	3.858	270	0.0061	−44.6	–
10% NiO	3.897	180	0.59	−59.1	158
30% NiO	3.891	–	–	−31.7	144
10% Co_3_O_4_	3.898	172	1.07	−52.7	164
30% Co_3_O_4_	3.909	265	7.25	−43.4	210
